# Estimation and Analysis of PM_2.5_ Concentrations with NPP-VIIRS Nighttime Light Images: A Case Study in the Chang-Zhu-Tan Urban Agglomeration of China

**DOI:** 10.3390/ijerph19074306

**Published:** 2022-04-03

**Authors:** Mengjie Wang, Yanjun Wang, Fei Teng, Shaochun Li, Yunhao Lin, Hengfan Cai

**Affiliations:** 1 Hunan Provincial Key Laboratory of Geo-Information Engineering in Surveying, Mapping and Remote Sensing, Hunan University of Science and Technology, Xiangtan 411201, China; wangmengjie@mail.hnust.edu.cn (M.W.); tengfei@mail.hnust.edu.cn (F.T.); lsc_gis@mail.hnust.edu.cn (S.L.); linyunhao@mail.hnust.edu.cn (Y.L.); chf@mail.hnust.edu.cn (H.C.); 2 National-Local Joint Engineering Laboratory of Geo-Spatial Information Technology, Hunan University of Science and Technology, Xiangtan 411201, China; 3 School of Earth Sciences and Spatial Information Engineering, Hunan University of Science and Technology, Xiangtan 411201, China

**Keywords:** multisource data, machine learning, PM_2.5_ concentration estimation, partial least squares

## Abstract

Rapid economic and social development has caused serious atmospheric environmental problems. The temporal and spatial distribution characteristics of PM_2.5_ concentrations have become an important research topic for sustainable social development monitoring. Based on NPP-VIIRS nighttime light images, meteorological data, and SRTM DEM data, this article builds a PM_2.5_ concentration estimation model for the Chang-Zhu-Tan urban agglomeration. First, the partial least squares method is used to calculate the nighttime light radiance, meteorological elements (temperature, relative humidity, and wind speed), and topographic elements (elevation, slope, and topographic undulation) for correlation analysis. Second, we construct seasonal and annual PM_2.5_ concentration estimation models, including multiple linear regression, support random forest, vector regression, Gaussian process regression, etc., with different factor sets. Finally, the accuracy of the PM_2.5_ concentration estimation model that results in the Chang-Zhu-Tan urban agglomeration is analyzed, and the spatial distribution of the PM_2.5_ concentration is inverted. The results show that the PM_2.5_ concentration correlation of meteorological elements is the strongest, and the topographic elements are the weakest. In terms of seasonal estimation, the spring estimation results of multiple linear regression and machine learning estimation models are the worst, the winter estimation results of multiple linear regression estimation models are the best, and the annual estimation results of machine learning estimation models are the best. At the same time, the study found that there is a significant difference in the temporal and spatial distribution of PM_2.5_ concentrations. The methods in this article overcome the high cost and spatial resolution limitations of traditional large-scale PM_2.5_ concentration monitoring, to a certain extent, and can provide a reference for the study of PM_2.5_ concentration estimation and prediction based on satellite remote sensing technology.

## 1. Introduction

In recent years, with the rapid development of China’s industrialization and urbanization, air quality problems have become increasingly intensified. In 2012, the Chinese government included PM_2.5_ concentration as an important pollution source indicator in the national environmental air quality standards [1,2]. PM_2.5_ can remain in the air for a long time, which will not only cause serious environmental problems, such as haze [3,4,5,6,7,8], but will also have a certain negative impact on meteorological changes, and it also has many health effects, such as premature mortality [9,10], hypertension [11], burden of disease [12,13], and health risks [14,15]. PM_2.5_ concentration monitoring is the key to the scientific management of PM_2.5_.

Traditional PM_2.5_ concentration monitoring methods include the manual particle sampling weight method, micro-oscillation balance method, and β-ray absorption method. These three ground monitoring methods have high accuracy and strong real-time performance. They are relatively used to common PM_2.5_ long-term monitoring methods, but the monitoring cost is too high, and the observation data from limited monitoring sites can only be used to characterize the PM_2.5_ concentration in the entire area. It is difficult to accurately monitor a large-scale geographic scene. Remote sensing data can be used to monitor the geographic phenomena of continuous ground surfaces for a long time. It has been widely used in PM_2.5_ concentration monitoring [16,17,18,19,20,21,22,23,24]. Kahn et al. [16] found that the particle size corresponding to the aerosol optical depth (AOD), obtained by the MISR inversion of the multiangle imaging spectrometer, was similar to the PM_2.5_ particle size, which proved the feasibility of establishing the correlation model between AOD and PM_2.5_. Li et al. [20] used satellite remote sensing parameters of AOD, fine mode fraction (FMF), planetary boundary layer height (PBLH), and atmospheric relative humidity (RH) to estimate PM_2.5_ concentrations and obtain high estimation accuracy. At the same time, a series of satellite images, such as Landsat, have also been used in PM_2.5_ concentration estimation [25,26]. In the above studies, satellite remote sensing technology is becoming more and more mature for daytime PM_2.5_ concentration estimation. However, it is difficult to monitor changes in PM_2.5_ concentration at night based on images obtained from visible light observations. At present, low-cost sensors are gradually being used in air quality monitoring. Relevant studies have shown that low-cost sensor sites with adequate monitoring conditions can provide high-quality PM_2.5_ concentration data, and they can effectively monitor the temporal and spatial changes of regional PM_2.5_ concentrations [27]. However, in some countries or regions, PM_2.5_ air pollution is not taken seriously, so the deployment of low-cost sensors on a large scale is still a long time away for developing countries.

Nighttime light images can effectively reflect the intensity of human activities, provide more spatial details of human society, and realize the time-series monitoring of the temporal and spatial dynamic changes of human social activities. Today’s nighttime light images have been widely used in socioeconomic and ecological environmental monitoring such as carbon emissions [28,29], GDP [30], poverty [31], city development [32,33], population density [34], and marine ships [28,29,30,31,32,33,34,35]. In addition to remote sensing images, commonly used for PM_2.5_ concentration estimation, nighttime light data have also been used to estimate PM_2.5_ concentrations at night. These nighttime light data are mainly from the Defense Meteorological Satellite Program’s Operational Linescan System (DMSP-OLP) [36,37,38] and National Polar-orbiting Visible Infrared Imaging Radiometer Suite (NPP-VIIRS) [39,40,41,42]. Wang et al. [40] used the day/night band (DNB) from radiation data of the Suomi National Polar-orbiting Partnership (S-NPP) satellite’s visible infrared imaging radiometer suite (VIIRS) to estimate PM_2.5_ concentration, and they found that nighttime light images can provide a good inversion of PM_2.5_ concentrations. The correlation coefficient R, between the estimated PM_2.5_ concentration and the measured PM_2.5_ concentration, is 0.67. Fu et al. [41] used data from the Day/Night Band (DNB) of the Visible Infrared Imaging Radiometer Suite (VIIRS) and hourly PM_2.5_ data, at 35 stations in Beijing, to develop a mixed-effects model to estimate nighttime PM_2.5_ concentrations. The results of cross-validation showed that the estimation accuracy of PM_2.5_ concentration in the four seasons was high, and the R^2^ of the model was greater than 0.80. Xu et al. [37] explored the influence of meteorological and social factors on PM_2.5_ concentrations, and their results showed that the nighttime light index was one of the main influencing factors of PM_2.5_ concentrations. Zhang et al. [42] combined meteorological data and satellite observation data, such as Luojia (LJ) 1-01 nighttime light images, to build a PM_2.5_ concentration estimation model. The LJ1-01 satellite is the first dedicated nighttime light remote sensing satellite in the world, and it launched in July 2018. The results showed that adding nighttime light image information can improve the performance of PM_2.5_ prediction models. The spatiotemporal distribution of PM_2.5_ concentration is a complex geographical phenomenon affected by multiple factors. It is difficult to explore the spatiotemporal relationship between nighttime light images and PM_2.5_ concentration from a smaller time scale. Long-term PM_2.5_ concentration estimation is an important part of air quality monitoring, but few studies have applied nighttime light images to seasonal and annual PM_2.5_ concentration estimations. The relationship between nighttime light images and long-term PM_2.5_ concentration temporal and spatial changes remains to be further studied.

The temporal and spatial distribution of PM_2.5_ concentration is a complex geographical phenomenon. Topographic and meteorological factors are important influencing factors for the temporal and spatial distribution of PM_2.5_ concentration [43]. Meteorological factors mainly depend on meteorological conditions, such as wind, precipitation, and temperature, to affect the regional PM_2.5_ concentration [44]. Wind acts on the temporal and spatial distribution of PM_2.5_ concentration by affecting air diffusion. Precipitation increases humidity and causes PM_2.5_ particles to clump together, unable to stay in the air, and fall to the ground. Changes in air temperature will affect the characteristics of atmospheric flow and, thus, the diffusion of PM_2.5_. Although topographic factors have less influence on the temporal and spatial distribution of PM_2.5_ concentration than meteorological factors [43], topographical factors such as altitude and slope affect the changes of PM_2.5_ concentration by changing the flow characteristics of air [45].

This paper analyzes the ability of nighttime light images to estimate seasonal and annual PM_2.5_ concentrations. This paper uses partial least squares to analyze the correlation between meteorological elements, terrain elements, nighttime light radiance, and PM_2.5_ concentration. Then, a multivariate linear and machine learning regression model for PM_2.5_ concentration estimation in the Chang-Zhu-Tan urban agglomeration was constructed, combined with the ground monitoring station data, to evaluate the accuracy of the model results, and finally, the spatial continuous distribution of PM_2.5_ concentration in the Chang-Zhu-Tan urban agglomeration was inverted.

## 2. Study Areas and Data Sources

### 2.1. Study Areas

**Figure 1:**
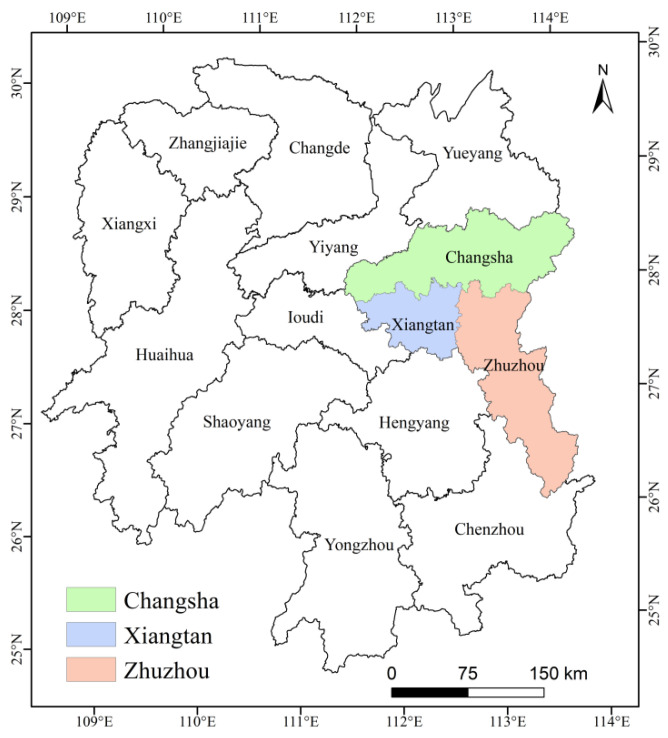
Location of the Chang-Zhu-Tan urban agglomeration.

The Chang-Zhu-Tan urban agglomeration is located in the middle-eastern part of Hunan Province (Figure 1). It has a mid-subtropical monsoon climate with four distinct seasons, short winters, long summers, and abundant rainfall. As the core growth pole of economic development in Hunan Province, the Chang-Zhu-Tan urban agglomeration industry has achieved rapid development in recent years [46]. At the same time, the problem of air pollution has become increasingly prominent, and the concentration of various air pollutants in the urban agglomeration remains high [47,48]. The air quality level ranks last in the province year round. Regional air pollution seriously affects public health and ecological safety, and the serious haze problem has also attracted great attention from all walks of life [49]. In recent years, the relevant air pollution control measures of the Chinese government have resulted in a significant decrease in the PM_2.5_ concentration in the Chang-Zhu-Tan urban agglomeration, effectively improving the air quality of the urban agglomeration [50].

### 2.2. Data Sources

The data for this research include PM_2.5_ concentration data, meteorological data, NPP-VIIRS nighttime light images, and Shuttle Radar Topography Mission (SRTM) digital elevation model (DEM) data in the Chang-Zhu-Tan urban agglomeration in 2015 and 2018.

**Figure 2:**
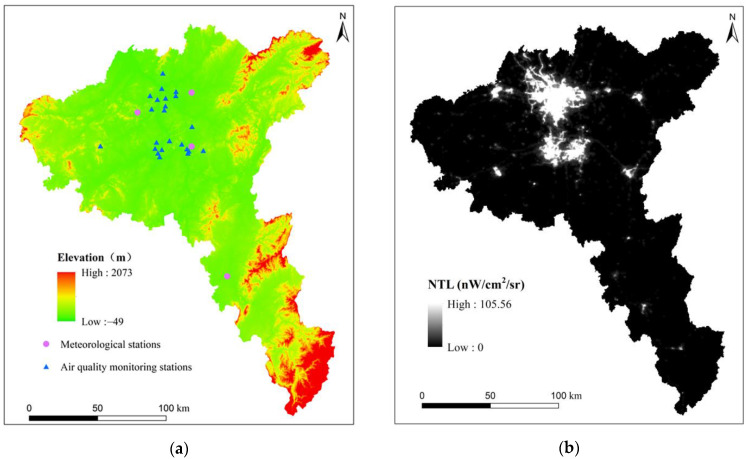
Datasets used in this study. (**a**) SRTM DEM data and spatial distribution of the monitoring stations; (**b**) NPP-VIIRS nighttime light (NTL) image.

PM_2.5_ concentration data: The PM_2.5_ concentration data used in this article came from the national urban air quality real-time release platform of the China Environmental Monitoring Station (CEMS. http://106.37.208.233:20035/ (accessed on 15 October 2019)). The quarterly and annual average PM_2.5_ concentrations were derived from the hourly monitoring data of 24 ambient air quality assessment monitoring points in the Chang-Zhu-Tan urban agglomeration (Figure 2a). In order to ensure the accuracy, continuity, and integrity of PM_2.5_ concentration measurement data, the Chinese government stipulates that, when automatic monitoring equipment is used for monitoring, the monitoring equipment needs to run continuously, 365 days a year. The daily average of PM_2.5_ concentration measurements requires at least 20 h of average concentration values or adoption time. The PM_2.5_ concentration measurement data in this paper are obtained by the continuous automatic monitoring method. The Chinese government stipulates that the PM_2.5_ automatic monitoring method with different principles can only be used to measure PM_2.5_ if it is consistent with the monitoring results of the manual gravimetric method. Therefore, the PM_2.5_ concentration measurement values used in this paper are subject to strict quality control and are effective.

Meteorological data: The meteorological data came from the National Meteorological Science Data Sharing Service Platform (NMSDSSP. http://data.cma.cn. (accessed on 15 October 2019)) and mainly include precipitation, temperature, relative humidity, and wind speed. The quarterly and annual average weather data came from the daily average values of meteorological stations in the Chang-Zhu-Tan urban agglomeration (Figure 2a). Meteorological factors have a great impact on the spatial distribution of PM_2.5_ in the Chang-Zhu-Tan urban agglomeration [47]. The meteorological information of the air quality monitoring stations comes from four ground meteorological stations. Since the air quality monitoring stations are distributed in the plain area and are concentrated near the four ground meteorological stations, the uniformity of meteorological factors in a small range is considered [51]. Therefore, it is feasible that the meteorological information of the air quality monitoring station comes from four ground meteorological stations in this study.

NPP-VIIRS nighttime light images were obtained from the Earth Observation Group (EOG). This article used the monthly data from NPP-VIIRS nighttime light images in 2015 and 2018, with a resolution of 500 m (Figure 2b). The monthly nighttime light image was composed of the cloudless nighttime light image of the month, which was the average radiation image. The monthly nighttime light images were also processed with stray light correction. The processed monthly NPP-VIIRS nighttime light images can effectively monitor the status quo of regional socioeconomic development [52,53,54]. Nighttime light images can effectively reflect the development status of human society and provide more spatial details of human activities [55,56].

SRTM DEM data: The DEM data of the experimental area came from the SRTM data of the U.S. Space Shuttle Endeavour. This dataset was based on the latest SRTM V4.1 data, through collation and splicing, to generate 90 m resolution DEM data (Figure 2a). Topography not only affects the spatial distribution of pollutant emissions by affecting the intensity of human activities but also has a profound impact on the diffusion of PM_2.5_, which is an important factor affecting the spatial distribution of PM_2.5_ [43,57].

## 3. Methods

### 3.1. Correlation Analysis between Remote Sensing Data and PM_2.5_ Concentration

Based on the theory of radiative transmission, the relationship model between nighttime light radiance and PM_2.5_ concentration in the near-surface layer can be constructed [40]. First, it is assumed that there is no change in the distribution of surface features (especially buildings and city lights) around the ground air quality monitoring site. Then, there is the nighttime light radiance, after reflection/scattering by various physical media from lights emitting upwards, from what is considered a Lambertian body, which is a constant with spatial differences [40]. Assuming negligible multiple scattering from aerosols, the nighttime light radiance reaching the sensor follows Beer’s law. Assuming that there is a good and stable aerosol extinction coefficient profile structure in the boundary layer at night, and PM_2.5_ is uniformly mixed at the effective height, the relationship between PM_2.5_ and nighttime light radiance can be established [40]. In this paper, the average value of nighttime light, 2 km around the environmental detection site, was extracted as its nighttime light radiance value.

Meteorological elements are important factors influencing the changes in PM_2.5_ concentration [44,58,59,60]. Wang et al. [58] discussed whether meteorological elements can affect PM_2.5_ concentrations and found that meteorological elements, such as humidity and air temperature, can affect the temporal and spatial distributions of PM_2.5_ concentrations. In addition, topographic elements affect the change in regional PM_2.5_ concentration to a certain extent [43,45,57]. He et al. [45] added the information extracted from DEM data to the PM_2.5_ estimation model, and the results showed that the model with topography, meteorology, and other elements can better estimate PM_2.5_ concentrations. Therefore, the PM_2.5_ concentration estimation model that takes into account the influence of multiple factors, such as weather and topography, at the same time can obtain higher-precision PM_2.5_ concentration simulation results. Therefore, the characteristic factors determined in this paper include nighttime light radiance I, elevation E, slope S, precipitation R, temperature T, relative humidity RHU, and wind speed W.

### 3.2. Selection of Characteristic Factors for the PM_2.5_ Concentration Estimation Model

The correlation analysis was carried out by constructing a partial least squares model of Factor Set A and PM_2.5_ concentration. The partial least squares method uses the algorithm of decomposing and screening the data information in the model, extracts the comprehensive variable with the strongest explanatory power for the dependent variable, and can calculate the importance of each factor. The partial least squares method can better solve the factor collinearity problem and obtain more objective and accurate factor importance results [61]. The variable importance in projection (VIP) value of partial least squares is used as the factor importance result [62], and the VIP value calculation formula is as follows:

(1) $$VIP_{j}=\sqrt{\frac{p\sum_{k=1}^{h}\left(c_{k}^{2}t_{k}^{’}t_{k}\right)w_{jk}^{2}}{\sum_{k=1}^{h}c_{k}^{2}t_{k}^{’}t_{k}}}$$

where: $VIP_{j}$ is the VIP value of the j-th variable; p is the number of variables participating in the analysis; h is the number of iteration calculations; $c_{k}^{2}t_{k}^{’}t_{k}$ is the interpretation of the dependent variable from the *k*-th independent variable mapping result interpretation degree; $w_{jk}^{2}$ is the weight of variable *j* in the *k*-th iteration.

### 3.3. Construction of the PM_2.5_ Concentration Estimation Model

Simple models have limitations in simulating complex geographic phenomena, with multiple factors, at high precision [63]. Zhang et al. [63] found that simple models cannot effectively estimate the spatial distribution of PM_2.5_ concentrations affected by multiple factors. In this paper, referring to the research results of Wang et al. [40], a multiple linear regression model was selected to construct the PM_2.5_ concentration estimation Model I of the Chang-Zhu-Tan urban agglomeration. There are 24 air quality monitoring stations in the Chang-Zhu-Tan urban agglomeration.

(2) $${\mathrm{PM}}_{2.5}=\beta_{0}+\beta_{1}X_{1}+\beta_{2}X_{2}+\cdots +\beta_{n}X_{n}$$

where: PM_2.5_ is the estimated PM_2.5_ concentration of the air quality monitoring site; $X_{1}$, $X_{2}$, and $X_{n}$ are the 1st, 2nd⋯nth estimated model factors, respectively; $\beta_{1}$, $\beta_{2}$, and $\beta_{n}$ are the regression coefficients of each model, respectively.

When there is no definite estimation method of PM_2.5_ concentration, the application of machine learning can extract key feature information to find the relationship between known datasets, and the machine model trained with a large amount of data can be used for accurate prediction. Machine learning methods have been increasingly used in socioeconomic parameter estimation and geographic phenomenon inversion, and there have also been related studies using machine learning methods for PM_2.5_ concentration estimation. Among them, there are many studies on the use of random forest models for PM_2.5_ concentration estimation [64,65,66], and other machine learning models are gradually applied to PM_2.5_ concentration estimation [67,68]. Based on the PM_2.5_ concentration data from ground stations and the known data of nighttime light radiance I, elevation E, slope S, precipitation R, temperature T, relative humidity RHU, and wind speed W, three machine learning PM_2.5_ concentration estimation models were constructed in this paper: random forest Model II, support vector machine Model III, and Gaussian process regression Model IV. These three models are more commonly used and more mature machine learning regression models. Each of them has some advantages. For example, support vector machines can solve machine learning problems with small samples and can find the nonlinear relationship between variables well. For unbalanced data sets, ensemble trees can balance errors to a certain extent. Gaussian process regression can quantify the prediction uncertainty in a principled way.

In this paper, the three machine learning estimation models were trained with multiple samples, and the fivefold cross-validation method was used to test model accuracy. Finally, the model parameters, when the goodness of fit (R^2^) of the model is the highest, are determined. According to the training results, the important parameters of the machine learning model with the highest R^2^ are selected (see Table 1). Among them, the parameter of random forest Model II is the minimum leaf size, and the parameters of Model III support vector machine and Model IV Gaussian process regression are the kernel function.

## 4. Results

### 4.1. Importance Analysis of PM_2.5_ Concentration Estimation Model Factors

To explore the influence of characteristic factors on the model estimation results, nighttime light radiance, elevation, slope, precipitation, air temperature, relative humidity, and wind speed were selected as Factor Set A. In addition, the more relevant feature factors from the Factor Set A were selected as Factor Set B. Finally, the precipitation, temperature, relative humidity, and wind speed of commonly used meteorological elements were selected from Factor Set A as Factor Set C.

In this paper, the partial least squares method was used to analyze the importance of the model factors. The VIP score of each factor obtained by the formula (1) determines the correlation between the factor and the PM_2.5_ concentration. The results showed that (Table 2) four meteorological factors (air temperature T, relative humidity RHU, precipitation R, and wind speed W) had high VIP scores. The mean VIP scores of quarterly and annual were 1.552, 0.795, 0.835, and 1.100, respectively. The air temperature T factor is the most important factor affecting the temporal and spatial distribution of PM_2.5_ concentration. There was a high correlation between nighttime light radiance I and PM_2.5_ concentration, with an average VIP score of 0.504. The topographic factors (elevation E and slope S) had a low correlation with the PM_2.5_ concentration, with average VIP scores of 0.320 and 0.304, respectively. Therefore, this paper selected temperature T, relative humidity RHU, precipitation R, wind speed W, and nighttime light radiance I as Factor Set B.

### 4.2. The Results and Accuracy Evaluation of the PM_2.5_ Concentration Estimation Model for the Chang-Zhu-Tan Urban Agglomeration

Based on the multiple linear regression model and three machine learning regression models, combined with the environmental monitoring site data of the Chang-Zhu-Tan urban agglomeration, model verification was carried out for the four seasons as well as annually (see Table 3 and Table 4).

Since the temporal and spatial distribution of PM_2.5_ concentration is a complex geographical phenomenon, the variation law of PM_2.5_ concentration, under the action of multiple factors, may be different in different time periods. Therefore, this paper considers selecting a variety of models to analyze the relationship between PM_2.5_ concentration and factors, in order to improve the estimation accuracy of PM_2.5_ concentration. The results showed that there were obvious differences in the estimation results of PM_2.5_ concentration models in different seasons, among which the PM_2.5_ concentration estimation model in spring had the worst results, and the R^2^ value was significantly lower than those from the other three seasonal and annual estimation models. The annual estimation model had the best effect, followed by the winter, summer, and autumn estimation models, which had similar effects.

There were also obvious differences in the estimation effects of different models. The multiple linear regression models had better estimation results for the seasonal PM_2.5_ concentration, while the machine learning model had better estimation results for the annual PM_2.5_ concentration. The number of sample points for the construction of seasonal and annual PM_2.5_ concentration estimation models was different. The number of sample points for seasonal PM_2.5_ concentration was small, only one-fourth of the number of annual PM_2.5_ concentration sample points, resulting in opposite results in the season and year for PM_2.5_ concentration estimation accuracy based on multivariate linear and machine learning models.

The effect of the estimation model of Factor Set B was obviously better than that of Factor Set C, indicating that adding nighttime light image information can effectively improve the performance of the estimation model. In addition, the estimation model effect of Factor Set A was better than that of Factor Set B, which also shows that adding topographic information can also effectively improve the model estimation ability.

**Figure 3:**
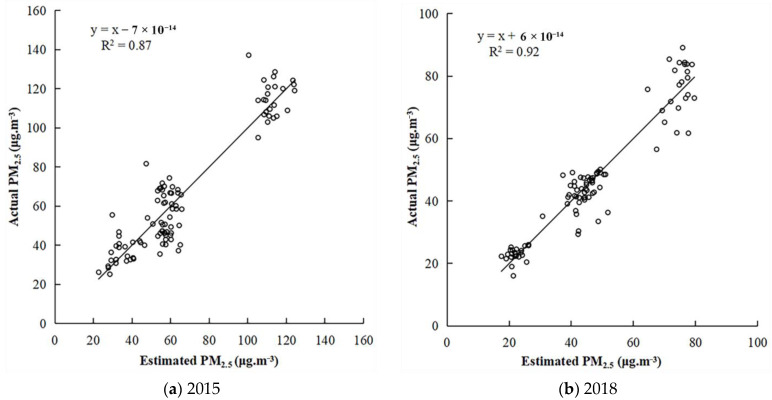
Scatter plots of estimated and actual PM_2.5_ concentrations.

At the same time, this paper established a scatter diagram between the annual estimated and actual PM_2.5_ concentrations (Figure 3). The results showed that there was a high correlation between the two, in which the R^2^ values in 2015 and 2018 were 0.87 and 0.92, respectively, indicating that there were good estimation results for the PM_2.5_ concentration.

### 4.3. Spatial Analysis of the PM_2.5_ Concentration in the Chang-Zhu-Tan Urban Agglomeration

**Figure 4:**
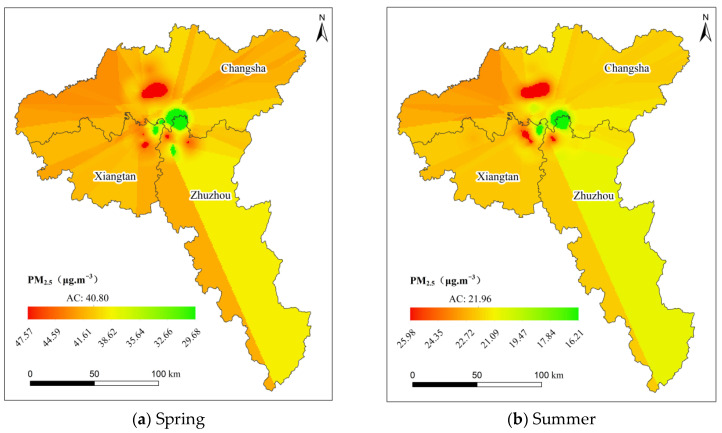

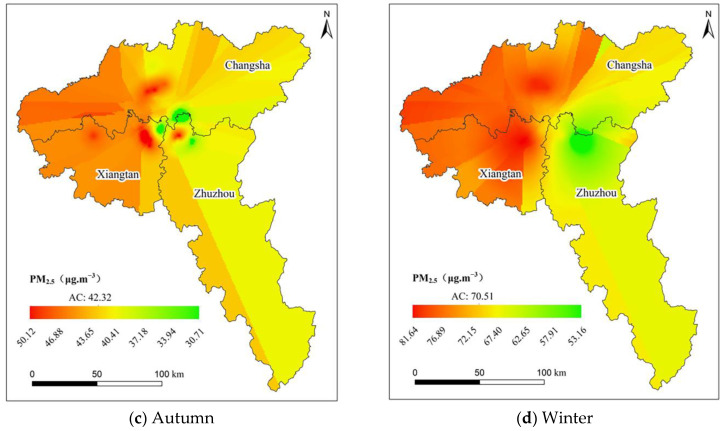
Inversion of seasonal PM_2.5_ concentration in 2018 in the Chang Zhu Tan urban agglomeration. AC means average PM_2.5_ concentration.

In this paper, kriging interpolation analysis was performed on the seasonal PM_2.5_ concentration of the Chang-Zhu-Tan urban agglomeration in 2018, and the continuous spatial interpolation of PM_2.5_ concentration was realized. The results are shown in Figure 4. According to the inversion results, the temporal and spatial distributions of seasonal PM_2.5_ concentrations in the Chang-Zhu-Tan urban agglomeration were analyzed. The results showed that the PM_2.5_ concentration of the Chang-Zhu-Tan urban agglomeration in winter was significantly higher than that in the other three seasons, with the lowest PM_2.5_ concentration in summer and similar PM_2.5_ concentrations in spring and autumn.

The study area is located in the subtropical monsoon region. The northerly wind prevails in the Chang-Zhu-Tan urban agglomeration in winter, the atmospheric structure is stable, and the meteorological conditions are not conducive to the diffusion of PM_2.5_ and other particles. The study area is prone to temperature inversion in winter, which makes PM_2.5_ particles gradually accumulate on the surface. In addition, the burning of a large amount of coal for heating in winter increases the PM_2.5_ concentration.

In summer, the southerly wind prevails, and the meteorological conditions are conducive to the diffusion of PM_2.5_ and other particles. In summer, strong winds are more likely to lead to the diffusion of PM_2.5_. In addition, it is rainy and humid in summer, and it is difficult for PM_2.5_ particles to stay in the air. The high temperature in summer makes it less likely for temperature inversion to occur, and the atmosphere is prone to convection, which is conducive to the diffusion of PM_2.5_ particles. Therefore, the concentration of PM_2.5_ is relatively high in winter and low in summer. At the same time, there are differences in the spatial distribution of PM_2.5_ concentrations. The PM_2.5_ concentration in the northwestern part of the Chang-Zhu-Tan urban agglomeration is relatively high, and the PM_2.5_ concentration in some central areas is low, which is significantly different from the adjacent areas.

## 5. Discussion

With the rapid development of industry and the increasing number of vehicles, the problem of air pollution is becoming increasingly serious [69]. Monitoring the spatial and temporal distribution of polluted gases is the key to solving the problem of air pollution. Among them, PM_2.5_ has always been one of the main air pollutants monitored by humans. At present, the model used by daytime remote sensing satellite technology for PM_2.5_ concentration estimation is relatively mature, and it can better perform spatial processing of large-scale PM_2.5_ concentrations. Human production and living activities greatly affect the temporal and spatial distributions of PM_2.5_ concentrations. Human social activities at night can reflect the intensity of human activities and reflect the state of human production, and living, to a certain extent. Therefore, this paper added nighttime light image information to PM_2.5_ concentrations. In the concentration estimation model, the results showed that the accuracy of the PM_2.5_ concentration estimation results has been somewhat improved, indicating that nighttime light images are of practical significance for PM_2.5_ concentration estimation.

In this paper, the partial least squares method was used to calculate the factor importance of the PM_2.5_ concentration. The partial least squares method can better solve the multicollinearity problem on the basis of retaining all factors, and the partial least squares method extracts, as much as possible, real PM_2.5_ concentration-related factor information to obtain a more objective and reliable correlation between factors and PM_2.5_ concentration. Compared with other factor analysis methods, the partial least squares method can calculate factor VIP scores on the basis of more effectively solving the multicollinearity problem.

**Figure 5:**
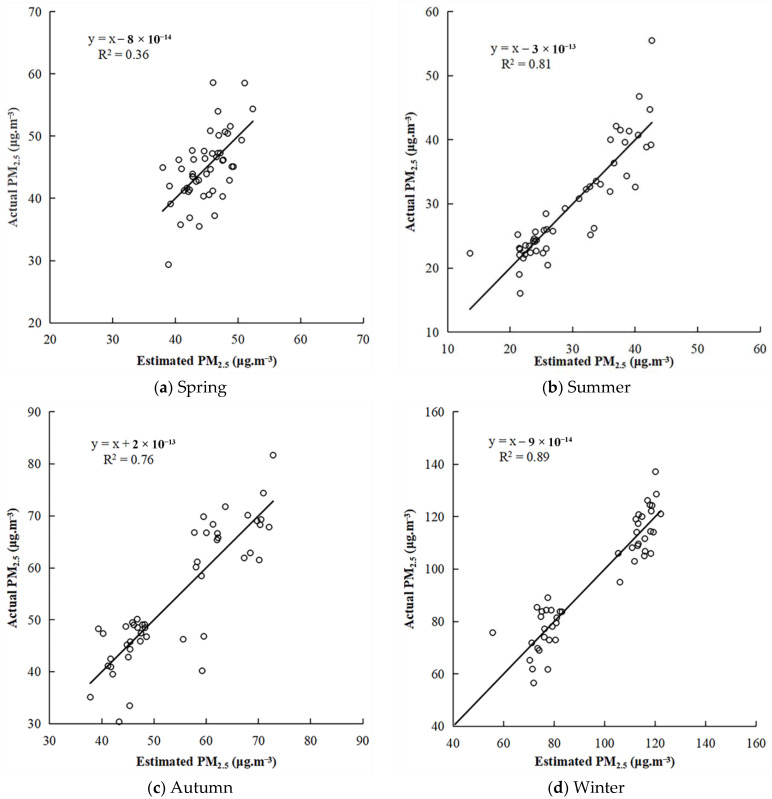
Scatter plots of estimated and actual PM_2.5_ concentrations in the four seasons.

In this paper, the multivariate linear model was used to obtain the estimated value of the seasonal PM_2.5_ concentration, and scatter plots (Figure 5) of the estimated value and the actual value of the PM_2.5_ concentration in the four seasons were constructed. The results showed that the estimated value and the actual value of the PM_2.5_ concentration in the four seasons was very close to y = x, indicating that the error distribution of the model, underestimating and overestimating PM_2.5_ concentration, was relatively balanced. The estimated R^2^ value of the PM_2.5_ concentration model in spring was significantly lower than that in the other three seasons, while the estimated R^2^ value of the PM_2.5_ concentration model in winter was significantly higher than that in the other three seasons, indicating that the model estimation accuracy had seasonality.

**Figure 6:**
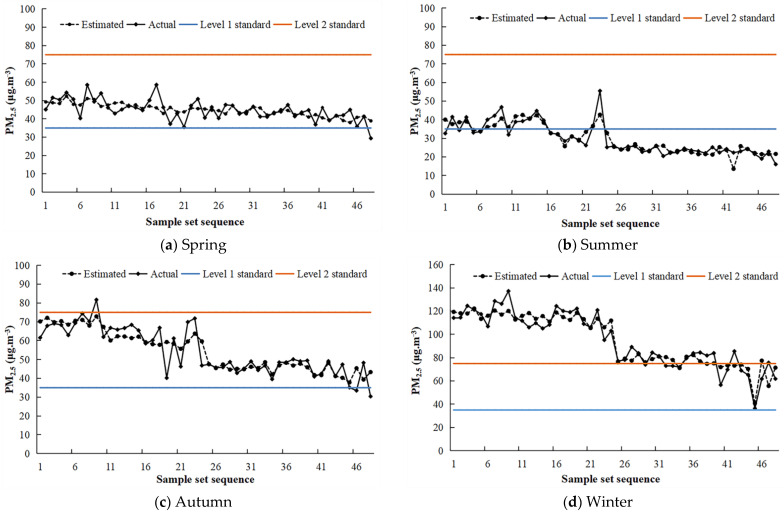
Comparison of the estimated and measured PM_2.5_ concentrations, given the sample set sequence. The blue line represents the Level 1 standard, and the orange line represents the Level 2 standard. The Level 1 standard refers to the 24-h average PM_2.5_ concentration lower than 35 µg·m^−3^. The Level 2 standard refers to the 24-h average PM_2.5_ concentration lower than 75 µg·m^−3^.

In addition, the spatial distribution of PM_2.5_ concentration is a complex geographic phenomenon, and the spatial characteristics of different air quality monitoring stations are different, resulting in obvious spatial differences in the accuracy of PM_2.5_ concentration estimation models. In this paper, the multivariate linear estimation model, with high estimation accuracy of seasonal PM_2.5_ concentration, was used to obtain the estimated PM_2.5_ concentration in the four seasons, and the estimated PM_2.5_ concentration in the four seasons was compared with the actual value (Figure 6). The results showed that the estimated and actual PM_2.5_ concentrations in the four seasons had similar trends, indicating that the overall effect of the model estimation was good, but there were still obvious local differences. The estimated value of the PM_2.5_ concentration, at some stations, was quite different from the actual value. To further analyze the spatial difference in model estimation accuracy, this paper also analyzed the actual error of PM_2.5_ concentration estimation at the stations. At the same time, it can be seen from the figure that the spring PM_2.5_ concentration of most air quality monitoring stations in the Chang-Zhu-Tan urban agglomeration was higher than the Level 1 standard but lower than the Level 2 standard. The summer PM_2.5_ concentration of most air quality monitoring stations was lower than the Level 1 standard, and the autumn PM_2.5_ concentration of air quality monitoring stations was similar to spring but significantly higher than the spring PM_2.5_ concentration. The PM_2.5_ concentration of air quality monitoring stations in winter was significantly higher than that of the other three seasons, and the winter PM_2.5_ concentration of most air quality monitoring stations was higher than the Level 2 standard.

**Figure 7:**
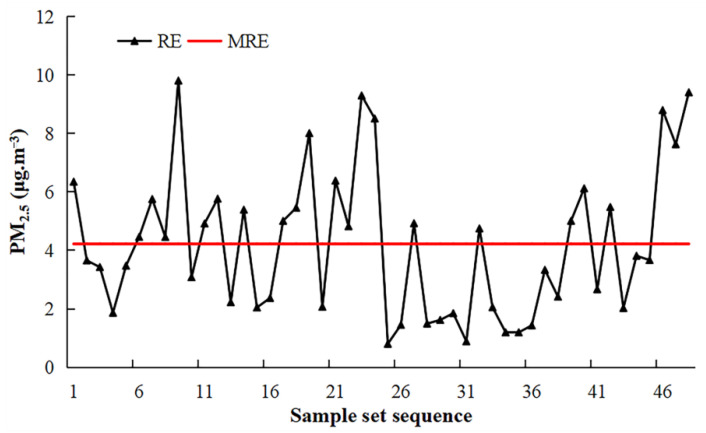
Error distribution of the estimated PM_2.5_ concentration, given the sample set sequence. RE refers to the real error of each station in the four seasons. MRE refers to the mean real error of 47 stations in the four seasons.

In this paper, a total of 48 air quality monitoring stations, in 2015 and 2018, were analyzed for the real error of PM_2.5_ concentration, and the average estimation errors of 48 stations in the four seasons were calculated (Figure 7). The results showed that the estimation error fluctuated greatly between stations, and there was an obvious uneven spatial distribution of model estimation errors. The total average error of 48 stations in the four seasons was 4.22 μg·m^−3^, and the estimation error of 23 stations was greater than the total average error. The spatial distribution of these 23 stations was further analyzed. Among them, 15 and 8 stations in 2015 and 2018, respectively, had estimation errors greater than the total average error, indicating that the estimation errors of PM_2.5_ concentrations, at stations in 2015, were relatively large.

**Figure 8:**
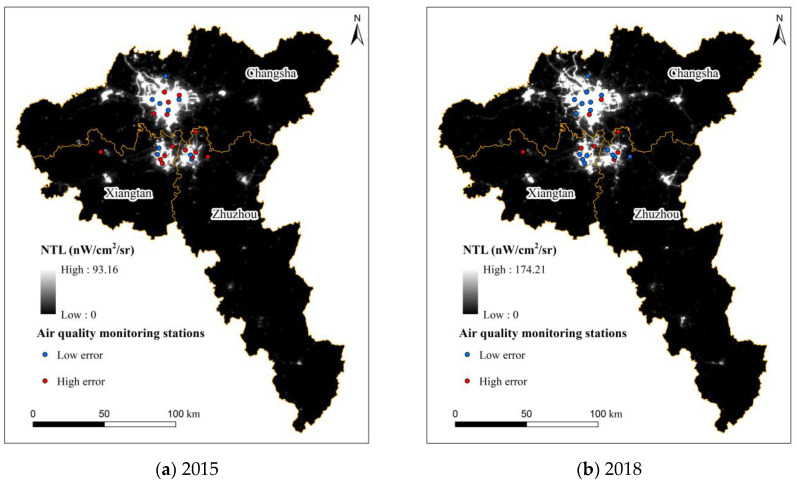
Spatial distribution of low and high-error stations.

Generally, an error higher than 4.22 μg·m^−3^ is a high error site, and an error lower than 4.22 μg·m^−3^ is a low error site. By analyzing the spatial locations of the 23 stations with large estimation errors, it can be found that the stations with high errors in 2015 and 2018 were mostly distributed in Xiangtan and Zhuzhou, and the economic development of these two cities was much slower than that of Changsha (Figure 8). The GDP of Changsha is 2.30 times that of the sum of the GDPs of Xiangtan and Zhuzhou, and the nighttime light area of Changsha is also much larger than that of Xiangtan and Zhuzhou. In addition, most stations distributed in dark areas at night had larger estimation errors, which was similar to the conclusion of Wang et al. [40]. The estimated models tended to underestimate PM_2.5_ concentrations in darker nighttime areas.

In this paper, a variety of estimation models for seasonal and annual PM_2.5_ concentrations were constructed based on nighttime light images, meteorological data, and topographic data. Except for spring, the models achieved high estimation accuracy, but further research is needed in terms of temporal and spatial resolution. In terms of temporal resolution, follow-up research should be more refined to the hourly scale. Nighttime light images, meteorological data, and topographic data can meet the requirements of this scale. However, in terms of spatial resolution, due to too few meteorological stations, the spatial resolution of meteorological conditions is limited. It is difficult to meet the high-precision inversion of PM_2.5_ concentrations. At the same time, the spatial resolution of the nighttime light images used in this paper is low, at only 500 m, and subsequent research should attempt to select higher spatial resolution images.

## 6. Conclusions

Based on multisource data and monitoring station PM_2.5_ concentration data, this paper constructed a variety of PM_2.5_ concentration estimation models for the Chang-Zhu-Tan urban agglomeration. The seasonal and annual PM_2.5_ concentrations of the Chang-Zhu-Tan urban agglomeration, in 2015 and 2018, were estimated, respectively, and the correlation between characteristic factors and PM_2.5_ concentrations was analyzed. The results showed that, in terms of the estimation results of the seasonal PM_2.5_ concentration model, the spring estimation results were the worst, and the winter estimation results were the best. Due to the increase in the number of samples in the annual PM_2.5_ concentration model, the estimation results of the machine learning model were better than the seasonal estimation results. In terms of the correlation of PM_2.5_ concentration, meteorological elements had a greater correlation with PM_2.5_ concentration, followed by nighttime light radiance, and terrain elements and PM_2.5_ concentration were the smallest.

This paper proposes a PM_2.5_ concentration estimation method based on multisource data. At the same time, there are some limitations in multisource data fusion and continuous surface PM_2.5_ concentration inversion, so further exploration is needed in subsequent research.

## Figures and Tables

**Table 1:** Important parameters of the various PM_2.5_ concentration estimation models based on machine learning.

Model Parameters	Spring	Summer	Autumn	Winter	Annual
Model II smallest leaf	12	4	12	12	12
Model III kernel function	Linear	Linear	Linear	Linear	Quadratic
Model IV kernel function	Exponential	Exponential	Matern 5/2	Exponential	Matern 5/2
Model parameters	Spring	Summer	Autumn	Winter	Annual

**Table 2:** VIP scores of different factors for the PM_2.5_ concentration estimation.

Factor	Spring	Summer	Autumn	Winter	Annual
*I*	1.138	0.464	0.366	0.302	0.249
T	1.381	1.507	1.658	1.465	1.748
RHU	1.157	1.449	0.530	0.662	0.178
W	0.508	0.985	0.979	0.986	0.719
*R*	0.943	0.526	0.742	1.384	1.907
E	0.414	0.257	0.322	0.442	0.164
S	0.723	0.249	0.175	0.283	0.091

**Table 3:** R^2^ values of the PM_2.5_ concentration estimation model in the Chang-Zhu-Tan urban agglomeration.

Model	Factor Set	Spring	Summer	Autumn	Winter	Annual
	Factor set A	0.36	0.81	0.76	0.89	0.82
Model I	Factor set B	0.31	0.79	0.75	0.88	0.82
	Factor set C	0.25	0.78	0.75	0.85	0.82
	Factor set A	0.17	0.65	0.72	0.79	0.90
Model II	Factor set B	0.16	0.66	0.72	0.80	0.92
	Factor set C	0.07	0.71	0.67	0.80	0.91
	Factor set A	0.23	0.69	0.69	0.77	0.88
Model III	Factor set B	0.20	0.55	0.66	0.75	0.90
	Factor set C	0.13	0.67	0.69	0.73	0.90
	Factor set A	0.08	0.64	0.54	0.73	0.89
Model IV	Factor set B	0.07	0.63	0.64	0.72	0.90
	Factor set C	0.06	0.67	0.63	0.72	0.92

**Table 4:** Root mean square errors of the PM_2.5_ concentration estimation model in the Chang-Zhu-Tan urban agglomeration.

Model	Factor Set	Spring	Summer	Autumn	Winter	Annual
	Factor set A	4.48	3.74	6.06	7.75	11.80
Model I	Factor set B	4.64	3.88	6.11	8.11	11.85
	Factor set C	4.85	3.94	6.15	8.91	11.90
	Factor set A	5.14	5.12	6.79	11.06	8.65
Model II	Factor set B	5.19	5.49	6.72	10.40	7.73
	Factor set C	5.50	4.64	7.10	10.69	8.25
	Factor set A	4.94	4.76	7.19	11.58	9.85
Model III	Factor set B	5.05	6.30	7.30	11.77	8.73
	Factor set C	5.34	4.98	6.90	12.45	8.75
	Factor set A	5.40	5.14	8.71	12.68	9.22
Model IV	Factor set B	5.44	5.69	7.57	12.30	8.67
	Factor set C	5.54	4.92	7.54	12.67	8.14

## Data Availability

No new data were created or analyzed in this study. Data sharing is not applicable to this paper.
